# Ohio State School of Veterinary Medicine

**Published:** 1895-07

**Authors:** 


### COMMENCEMENT EXERCISES.

### Ohio State School of Veterinary Medicine.

The Ohio State School of Veterinary Medicine connected with the Ohio State University at Columbus, Ohio, held their commencement exercises on June 11th and 12th. The following list of graduates received the college degree of D.V.M.: Frank Alexander Hamilton, Pennsylvania; Rollo Nooman Mead, Wood County, Ohio; Norman C. Powell, Columbiana County, Ohio; Frederick Priest, Licking County, Ohio.

